# CD38 Defines a Subset of B Cells in Rainbow Trout Kidney With High IgM Secreting Capacities

**DOI:** 10.3389/fimmu.2021.773888

**Published:** 2021-11-30

**Authors:** Diana Martín, Pedro Perdiguero, Esther Morel, Irene Soleto, J. German Herranz-Jusdado, Luis A. Ramón, Beatriz Abós, Tiehui Wang, Patricia Díaz-Rosales, Carolina Tafalla

**Affiliations:** ^1^ Animal Health Research Center, Instituto Nacional de Investigación y Tecnología Agraria y Alimentaria, Consejo Superior de Investigaciones Científicas (CISA-INIA-CSIC), Madrid, Spain; ^2^ Scottish Fish Immunology Research Centre, School of Biological Sciences, University of Aberdeen, Aberdeen, United Kingdom

**Keywords:** teleosts, B cells, CD38, IgM, plasmablasts, kidney

## Abstract

CD38 is a multifunctional molecule that functions both as a transmembrane signaling receptor and as an ectoenzyme with important roles in cell adhesion, calcium regulation and signal transduction. Within the B cell linage, CD38 is expressed in diverse murine B cell subsets, with highest levels in innate B cell subpopulations such as marginal zone (MZ) B cells or B1 cells. In humans, however, CD38 is transiently expressed on early lymphocyte precursors, is lost on mature B cells and is consistently expressed on terminally differentiated plasma cells. In the present work, we have identified two homologues of mammalian CD38 in rainbow trout (*Oncorhynchus mykiss*), designating them as CD38A and CD38B. Although constitutively transcribed throughout different tissues in homeostasis, both CD38A and CD38B mRNA levels were significantly up-regulated in head kidney (HK) in response to a viral infection. In this organ, after the generation of a specific monoclonal antibody (mAb) against CD38A, the presence of CD38A^+^ populations among IgM^+^ B cells and IgM^-^ leukocytes was investigated by flow cytometry. Interestingly, the percentage of IgM^+^CD38A^+^ B cells increased in response to an *in vitro* stimulation with inactivated *Aeromonas salmonicida*. Finally, we demonstrated that HK IgM^+^CD38A^+^ B cells had an increased IgM secreting capacity than that of cells lacking CD38A on the cell surface, also showing increased transcription levels of genes associated with B cell differentiation. This study strongly suggests a role for CD38 on the B cell differentiation process in teleosts, and provides us with novel tools to discern between B cell subsets in these species.

## Introduction

Different B cell subsets are defined in mammals. These include follicular (FO) B2 cells (conventional B cells), B1 cells and marginal zone (MZ) B cells. FO B2 cells, which constitute the largest B cell compartment in adult mammals, are generally activated in response to thymus dependent (TD) antigens within the lymphoid follicles and trigger the formation of germinal centers (GCs). Within these sites, B cells receive co-stimulatory signals from T follicular helper cells (Tfh) and specialized follicular dendritic cells (fDCs) and differentiate to antibody-secreting cells (ASCs), plasmablasts and eventually terminally-differentiated plasma cells (PCs). Throughout this differentiation process some cells become memory B cells. In mammals, during this follicular differentiation, isotype switching recombination takes place to remove the variable H chain segment (VDJ) of IgM and associate it with a different constant (CH) region, consequently secreting Ig isotypes with increased affinity such as IgG, IgE, or IgA (1). In parallel, the genes encoding the variable domains of the heavy and light chains undergo a high rate of point mutations by a process called somatic hypermutation (SHM). SHM results in an increased diversity of the antibody pool after which only B cells with higher affinity are selected by the antigen. This diversification of Ig genes is critical for the generation of an adequate specific immune protection (2). In contrast to conventional B2 cells, MZ B cells and B1 cells are usually considered elements of the innate immune system, being globally designated as innate B cells (3). These innate B cell populations mount extrafollicular immune responses outside the GCs in the absence of cognate T cell cooperation. They are normally activated by thymus independent (TI) antigens that stimulate the B cell receptor (BCR) and are simultaneously recognized by innate receptors expressed in these B cells (4). MZ B cells seem to specifically recognize blood-borne pathogens in the spleen (5), while B1 cells recognize pathogens in the early stages of the immune response and produce natural IgM antibodies with low affinity and wide reactivity until specific B2 cells respond (6). In mammals, B1 cells are mainly found in the peritoneal and pleural cavities, mucosal surfaces and spleen, but are absent or scarce in lymph nodes and peripheral blood (7, 8).

Teleost fish do not possess lymph nodes and do not develop conventional GCs. Interestingly, several recent studies have revealed many similarities between mammalian B1 cells and teleost B cells, including a strong phagocytic capacity (9, 10), the expression of B1-specific markers such as CD9 and CD5 (11, 12), the transcription of a wide range of pattern recognition receptors (PPRs) or the low IgD and high IgM surface expression (12). The resemblance of teleost B cells to mammalian B1 cells, along with the fact that no cognate sites where B and Tfh cells interactions are organized in teleosts during the immune response, strongly suggests that teleost B responses are analogous to mammalian extrafollicular IgM responses. In agreement with this hypothesis, is the fact that teleost fish lack high affinity Igs such as IgG, IgE, or IgA and therefore do not undergo class switch recombination (CSR).

Two different ADP-ribosyl cyclase/cyclic ADP-ribose hydrolase proteins have been described in vertebrates to date. The first one is encoded by *CD38* and the second one is encoded by the bone marrow stromal antigen 1 gene (*BST1*). CD38 is a 45-kDa type II glycoprotein with multiple roles, as an ectoenzyme, a transmembrane receptor or an adhesion molecule. Thus, CD38 is a bifunctional enzyme capable of converting NAD^+^ into Ca^2+^-mobilizing agent cADPR, and subsequently hydrolyzing cADPR to ADPR (13). These reaction products are essential for the regulation of intracellular Ca^2+^, an ancient and universal cell signaling system that conditions many immune cell functions. As a receptor, CD38 binds the specific ligand CD31, a surface molecule mainly expressed by endothelial cells (14), thereby regulating cell adhesion. Additionally, it has been documented that CD38 can establish strong associations with professional signaling complexes of different cell lineages (i.e., CD3/TCR in T lymphocytes, BCR/CD19/CD21 in B lymphocytes and CD16/CD61 in NK cells). Through these interactions, CD38 plays a critical role in transducing activating signals in these immune cell populations (15). Although first identified as a marker for thymic T cells (16) and later on for B cells (17), it is now known that is a widely distributed molecule in both hematopoietic and non-hematopoietic cells such as brain neurons, prostate epithelial cells or pancreatic cells [reviewed in (18)]. Among hematopoietic cells, CD38 is broadly expressed in monocytes, myeloid cells, B and T cells, NK cells, platelets [reviewed in (18)]. In most of these cells, the expression of CD38 is regulated by stimulation or defines particular subsets of cells with specific functions. Hence, for example, in humans, CD38 expression has been reported in circulating monocytes but not in macrophages (19). Nevertheless, monocytes up-regulate further CD38 expression levels upon stimulation with interferon γ (IFN-γ) but not in response to LPS or tumor necrosis factor α (TNF-α) (19). Within the T cell linage, high CD38 expression levels have been reported in specific T cell receptor (TCR)^+^CD4*^-^* CD8*^-^* thymocytes with unique regulatory functions (20), or in a subset of activated T cells with reduced proliferating capacities but improved potential to produce cytokines (21). Similarly, CD38 marks a subset of mature human NK cells that display enhanced killing and IFN-γ secretion capacities (22). Within the B cell lineage, different patterns of expression are found in mice and humans. In mice, immature B cells emerging from bone marrow express CD38. In the spleen, transitional 2 lymphocytes (T2) express more CD38 than transitional 1 lymphocytes (T1) or mature naive FO B cells, whereas MZ B cells have higher CD38 expression levels. Nonetheless, B1 cells found in the peritoneal cavity have the highest expression of CD38 on their surface in mice [reviewed in (23)]. In contrast, in humans, terminally differentiated plasma cells express the highest levels of surface CD38 (24). Nonetheless, to date, the role of CD38 during plasma cell differentiation is still unclear in mammals.

In the current work, we have identified two homologues of mammalian CD38 in rainbow trout, designating them as CD38A and CD38B. We have analyzed their levels of transcription throughout diverse tissues and in response to a viral infection in the head kidney (HK). The HK is the main hematopoietic tissue in teleost fish, as well as the main B cell maturation site (25). Additionally, we have produced a specific monoclonal antibody (mAb) against CD38A, and used it in flow cytometry to differentiate between HK IgM^+^ B cells that express CD38A on the cell surface (IgM^+^CD38^+^ B cells) and those that do not (IgM^+^CD38^-^ B cells). Interestingly, the capacity of IgM^+^CD38^+^ B cells to secrete IgM was significantly higher than that of the IgM^+^CD38^-^ B cell population. In concordance, the transcription levels of genes related to B cell differentiation to plasmablasts/PCs were also higher in B cells expressing CD38A on the cell surface. Altogether, our results point to CD38A as a differentiation marker for B cells in rainbow trout, whereas the antibody produced in this work constitutes a novel tool to help us differentiate specific B cell subsets in teleosts.

## Material and Methods

### *In Silico* Identification and Analysis of CD38 Homologues in Rainbow Trout

Using human CD38 and BST1 as queries and the Blastp software, all potential genes encoding ADP-ribosyl cyclase/cyclic ADP-ribose hydrolase proteins from *Oncorhynchus mykiss* were identified along the reference genome Omyk_1.0 obtained from the RefSeq genome database. In the same way, several protein sequences homologous to human CD38 and BST1 from several species were retrieved from the genomes present in the RefSeq database. In order to analyse the evolution of these genes, a selection of proteins from several species covering different classes were included in a multiple protein alignment using the ClustalW software. The alignment was used for the construction of a phylogenetic tree using maximum likelihood, testing the tree with a bootstrap method using 1,000 replications. All steps were implemented in the MEGA X software (26). The ADP-ribosyl cyclase/cyclic ADP-ribose hydrolase proteins identified in *Branchiostoma belcherii* and *B. floridae* were included as an outgroup and used for rooting purposes.

The synteny analysis was performed using the information related to *CD38* and *BST1* and their neighbouring genes available in the RefSeq genomes. For this purpose, the proteins from key species were identified in the phylogenetic tree previously constructed. The selection of species included *Petromyzon marinus* as the most ancient species, three different chondrichthye species (*Callorhinchus milii*, *Scyliorhinus canicula* and *Amblyraja radiate*) and a group of ancient fish with a key position in fish evolution (*Latimeria cholumnae*, *Erpetoichthys calabaricus, Acipenser ruthenus* and *Lepisosteus oculatus*). The synteny analysis also included the genomic information from additional teleost fish (*Anguilla anguilla, Scleropages formosus, Paramormyrops kingsleyae*, *Danio rerio, Esox lucius*, *Oreochromis niloticus*, *Oryzias latipes*, *Perca flavescens* and *Takifugu rubripes*), together with a group representing salmonids (*Oncorhynchus mykiss*, *Salmo salar*, *Salmo trutta* and *Salvelinus alpinus*). Finally, several tetrapods (*Homo sapiens*, *Mus musculus*, *Gallus gallus, Crocodylus porosus, Chrysemys picta, Anolis carolinensis* and *Xenopus tropicalis*) were also included in the analysis. The information related to the 5-6 coding neighbouring genes at both sides of the genes of interest were analysed using the NCBI genome data viewer tool (https://www.ncbi.nlm.nih.gov/genome/gdv/), extracting the direction relative to *CD38* and the annotation assigned by RefSeq. The annotation was revised using the Blastp software, comparing the coding protein sequence from neighbouring genes as queries using UniProtDB as a reference database. To obtain the synteny images, the gene information was shown together with a phylogenetic tree based on the one originally constructed by Berthelot et al. (27).

The potential secondary and three-dimensional (3D) structure of *O. mykiss* ADP-ribosyl cyclase/cyclic ADP-ribose hydrolase proteins was analyzed using the Phyre2 software (28). For this purpose, the amino acid sequences were examined using the normal mode method by which the sequence regions encoding alpha helixes, beta strands or transmembrane helixes were identified. At the same time, the sequences were compared with different domains corresponding to fold libraries contained in the Phyre2 database, selecting the most confident structure as a template for the 3D model.

### Fish Maintenance

Healthy rainbow trout (*O. mykiss*) of different sizes were obtained from *Piscifactoria Cifuentes* (Cifuentes, Guadalajara, Spain) and maintained at the animal facilities of the Animal Health Research Centre (CISA-INIA-CSIC, Spain) in an aerated recirculating water system at 14°C, with a 12:12 h light: dark photoperiod. Fish were fed twice a day with a commercial diet (Skretting, Spain). Prior to any experimental procedure, fish were acclimatized to laboratory conditions for at least 2 weeks. During this period no clinical signs of disease were ever observed.

### Evaluation of CD38A and CD38B Transcription in Rainbow Trout Tissues

Rainbow trout of approximately 10-15 cm maintained at CISA-INIA-CSIC were euthanized by benzocaine (Sigma Aldrich) overdose. Spleen, head and posterior kidney, thymus, skin, gills, foregut, pyloric caeca, stomach, midgut, hindgut, liver, brain, heart, gonad and adipose tissue were collected and placed in TRIzol (Invitrogen) after having performed a transcardial perfusion using teleost Ringer solution pH 7.4 with 0.1% procaine in order to remove all the circulating blood from the tissues (29, 30).

Total RNA was extracted from tissue samples using a combination of TRIzol and RNAeasy Mini kit (Qiagen) previously described (11). In summary, samples were mechanically disrupted in 1 ml of TRIzol using a disruption pestle. Then, 200 µl of chloroform were added and the suspension centrifuged at 12,000 x *g* for 15 min. The clear upper phase was recovered, mixed with an equal volume of 100% ethanol and immediately transferred to RNAeasy Mini kit columns. The procedure was then continued following manufacturer’s instructions, performing on-column DNase treatment. Finally, RNA pellets were eluted from the columns in RNase-free water, quantified in a Nanodrop 1000 spectrophotometer (Thermo Scientific) and stored at -80°C until use. Two µg of RNA were used to obtain cDNA in each sample using the Bioscript reverse transcriptase (Bioline Reagents Ltd) and oligo (dT)_12-18_ (0.5 µg/ml) following manufacturer´s instructions. The resulting cDNA was diluted in a 1:5 proportion with water and stored at -20°C.

To evaluate the levels of transcription of the two CD38 homologues, real-time PCRs were performed in a LightCycler 96 System instrument (Roche) using SYBR Green PCR core Reagents (Applied Biosystems) and specific primers (shown in **Table S1**). The efficiency of the amplification was determined for each primer pair using serial 10-fold dilutions of pooled cDNA, and only primer pairs with efficiencies between 1.95 and 2 were used. Each sample was measured in duplicate under the following conditions: 10 min at 95°C, followed by 40 amplification cycles (15 s at 95°C and 1 min at 60°C). A melting curve for each PCR was determined by reading fluorescence every degree between 60°C and 95°C to ensure only a single product had been amplified. The expression of individual genes was normalized to relative expression of trout EF-1α and the expression levels were calculated using the 2^-ΔCt^ method, where ΔCt is determined by subtracting the EF-1α value (**Table S2**) from the target Ct as previously described (31, 32). Negative controls with no template and *minus* reverse transcriptase controls were included in all the assays.

### Leukocyte Isolation

Rainbow trout of approximately 20-25 cm were euthanized by benzocaine overdose and spleen, HK and gills removed. Tissue suspensions were prepared using 100 µm nylon cell strainers (BD Biosciences) and Leibovitz’s medium (L-15, Gibco) containing 100 I.U./ml penicillin and 100 µg/ml streptomycin (P/S, Life Technologies), 10 IU/ml heparin and 2% fetal calf serum (FCS, Thermo Fisher Scientific). Cell suspensions were then placed onto 30/51% Percoll (GE Healthcare) density gradients and centrifuged at 500 x *g* for 30 min at 4°C. Leukocytes were also isolated from peripheral blood. For this, blood obtained from the caudal vein was diluted 10 times with L-15 medium containing P/S, 10 U/ml heparin and 5% FCS and placed onto 51% Percoll density gradients and centrifuged at 500 x *g* for 30 min at 4°C. In all cases, the interface cells were collected and washed with L-15 supplemented with antibiotics and 2% FCS. The viable cell concentration was determined by Trypan blue (Sigma-Aldrich) exclusion and cells were resuspended in L-15 with 5% FCS at a concentration of 2 x 10^6^ cells/ml.

### Evaluation of CD38A and CD38B Transcription in IgM^+^ B Cells

The constitutive levels of transcription of the two CD38 homologues were studied in IgM^+^ B cells from HK and spleen. For this, leukocyte suspensions from the two tissues were washed in FACS staining buffer (phenol red-free L-15 medium supplemented with P/S and 2% FCS) and incubated with a mAb specific for rainbow trout IgM [1.14 mAb mouse IgG_1_ coupled to R-phycoerythrin (R-PE), 1 µg/ml] (33). After 30 min of incubation at 4°C, cells were washed with FACS staining buffer and the YO-PRO dye (0.05 μM) added to the suspension for dead cell exclusion. Lymphoid (small, low complexity) IgM^+^ YO-PRO^-^ (live) cells were then isolated in a FACSAria™ III sorter (BD Biosciences) equipped with BD FACSDiva™ software (BD Biosciences). The purity of the sorted population (above 98%) was confirmed in a FACS Celesta flow cytometer (BD Biosciences).

DNase I-treated total RNA was reverse transcribed directly from FACS sorted populations using the Power SYBR Green Cells-to-Ct Kit (Invitrogen) following the manufacturer’s instructions. For comparative purposes, RNA was also isolated from the RTS11 rainbow trout macrophage-monocyte cell line (34). Real-time PCR was performed using SYBR Green PCR core Reagents (Applied Biosystems) using specific primers (**Table S1**) and following the manufacturer´s instructions as described previously (30).

### VHSV *In Vivo* Infection

Rainbow trout of approximately 6-8 cm maintained at CISA-INIA-CSIC were challenged with viral hemorrhagic septicemia virus (VHSV) by bath as previously described (35). Briefly, fish were transferred to 4 l of a viral solution containing 5 x 10^5^ TCID_50_/ml of the VHSV strain 0771. After 1 h of viral adsorption with strong aeration at 14°C, each experimental group was transferred to an individual water tank. Mock-infected groups were treated in the same way in the absence of virus. At days 1, 3 and 7 post-infection, six trout from each group were sacrificed by over-exposure to benzocaine. HK were sampled and placed in TRIzol for RNA isolation. Total RNA was extracted from tissue samples as described above.

### Production and Characterization of an Anti-CD38A Antibody

The nucleotide sequence corresponding to the extracellular domain of CD38A (**Figure S1**) was synthetized and subcloned into the E3 expression vector (Abyntek) together with an N-terminal 6 x histidine tag. The recombinant plasmid was transformed into BL21 cells and a kanamycin-resistant single positive colony was then incubated at 37°C in Luria-Bertani (LB) media. When the OD600 reached 0.6, 0.1 mM of isopropyl β-D-thiogalactoside (IPTG, Sigma Aldrich) was added to induce protein production. After 16 h, cells were harvested, lysed by sonication and dissolved using urea. Thereafter, CD38A was obtained through the use of Nickel columns (Sigma Aldrich). The CD38A-containing fractions were pooled, refolded and used to immunize three independent mice. Animals were immunized intravenously (i.v.) at days 0, 15, 30, and 45. Mice were sacrificed 3 d after the last immunization, and splenocytes isolated. Generation of hybridomas by fusion of mouse splenocytes with SP2 myeloma cells, isolation of clones, and purification of specific anti-trout mAbs were performed as previously described (36). The recombinant CD38A protein was used to test the specificity of the antibodies by ELISA following methods previously described (37).

### Western Blot

The extraction of proteins (soluble proteins and cell membrane proteins) was carried out as described by Bouchet-Bernet et al. (38) with minimal modifications. Briefly, fresh HK tissue obtained from 20-25 cm rainbow trout was pulverized with a Polytron in 4 ml of Buffer SB [10 mM Tris-HC1, 0.5 mM dithiothreitol, 1.5 mM EDTA, 10% (v/v) glycerol, and 1 tablet of Mini Protease Inhibitor Cocktail (Roche) per 50 ml of buffer, pH 7.4] at 4°C. The sample was then centrifuged at 10,000 x *g* for 20 min at 4°C. The supernatant corresponding to the soluble fraction was removed, aliquoted and stored to -80°C until use. The pellet was dissociated by pipetting up and down with 4 ml of Buffer MB [20 mM Tris-HC1, 125 mM NaC1, 1% (v/v) Triton X-100 and 1 tablet of Mini Protease Inhibitor Cocktail per 50 ml of buffer, pH 7.4]. Thereafter, the sample was centrifuged at 10,000 x *g* for 20 min at 4°C and the supernatant corresponding to the cell membrane fraction removed, aliquoted and stored to -80°C until use. These kidney protein lysates as well as the recombinant CD38A protein were separated by sodium dodecyl sulfate-polyacrylamide gel electrophoresis (SDS-PAGE) and electrotransferred to a nitrocellulose membrane which was incubated with the anti-trout CD38A (final concentration 2 ug/ml) following standard methods (39). The antigen-antibody complex was developed by using anti-mouse streptavidin-peroxidase and the ECL reagent (GE Healthcare) following the manufacturer´s instructions.

### Flow Cytometry

HK, blood, spleen and gill leukocytes were stained with anti-trout IgM [1.14 mAb mouse IgG1 coupled to R-phycoerythrin (R-PE), 0.25 µg/ml] and anti-trout CD38A [mAb mouse IgG2b coupled to FITC, 5 µg/ml] for 1 h at 4°C in the dark in staining buffer. After this time, cells were washed twice with staining buffer. The cell viability was checked by addition of 4’,6-diamine-2’-phenylindole dihydrochlorid (DAPI 0.2 µg/ml). Cells were analysed on a FACS Celesta flow cytometer (BD Biosciences) equipped with BD FACSDiva™ software. Flow cytometry analysis was performed with FlowJo V10 (TreeStar).

In some experiments, HK leukocytes were exposed to different stimuli prior to studying the distribution of CD38A. For this, leukocytes in L-15 medium supplemented with antibiotics and 5% FCS were dispensed into 24-well plates (Nunc) and exposed to *Aeromonas salmonicida* previously inactivated for 1 h at 65°C (2 x 10^6^ bacteria/ml) or VHSV previously inactivated for 30 min at 56°C (1 x 10^6^ TCID_50_/ml). Cells incubated with media alone were also included. After 72 h of incubation at 20°C, cells were stained with anti-IgM and anti-CD38A and analysed by flow cytometry as described above.

To test whether the mAb raised against CD38A was capable of detecting CD38A on the cell surface by flow cytometry spleen leukocytes were stained with anti-CD38A coupled to PECy5 (20 μg/ml) and the binding specificity was assessed by flow cytometry blocking the antigen recognition site with the recombinant protein used for the immunization at different peptide:mAb ratios (200:1. 100:1 and 50:1).

In order to study the amount of intracellular IgM within IgM^+^CD38A^+^ and IgM^+^CD38^-^ B cells, an intracellular IgM staining was carried out on HK leukocytes. For this, cells were first stained with anti-trout IgM coupled to APC (0.3 µg/ml) and anti-trout CD38 coupled to FITC (5 μg/ml) for 30 minutes. The cells were then fixed for 15 min with 4% PFA, permeabilized with 0.05% saponin and stained again with anti-trout IgM coupled to APC (0.3 µg/ml).

### Sorting of CD38A^+^IgM^+^ B Cells

HK leukocytes were stained with anti-trout IgM [1.14 mAb mouse IgG1 coupled to allophycocyanin, 0.5 µg/ml] and anti-trout CD38A [mAb mouse IgG2b coupled to FITC, 5 µg/ml] for 1 h at 4°C in the dark in staining buffer. Following several washing steps, cells were resuspended in staining buffer and IgM^+^CD38A^+^, IgM^+^CD38A^-^ and IgM^-^CD38A^+^ cells isolated by flow cytometry using a BD FACSAria III cell sorter based on the fluorescence emitted by the anti-IgM and anti-CD38A antibodies. Approximately 50,000 isolated IgM^+^CD38A^+^, IgM^+^CD38A^-^ B cells and IgM^-^CD38A^+^ cells were seeded in 96-well plates and incubated at 20°C for 3 days. After this time, supernatants were collected to determine total IgM concentration in supernatants by ELISA. Additionally, 50,000 IgM^+^CD38A^+^ and IgM^+^CD38A^-^ B cells were collected for subsequent RNA isolation and analysis of immune gene transcription using the Power SYBR Green Cells-to-Ct Kit as described above.

### ELISA

An ELISA was used to assess total IgM levels in supernatants obtained from sorted HK IgM^+^ B cell populations. For this, 96-well ELISA plates were coated overnight with 100 µl of 2 µg/ml mouse anti-trout Ig mAb (1.14). Wells were then blocked with 100 µl of 1% BSA in 0.3%Tween 20 PBS with for 1 h at RT. Plates were washed 3 times with 0.3% Tween 20 PBS and supernatants added to the wells. Samples were then incubated for 1 h at RT and washed 3 times in 0.3% Tween 20 PBS. Then, 50 µl of biotinylated 1.14 mAb (1 µg/ml) diluted in blocking buffer were added to the wells and samples incubated for 1 h at RT. After three washing steps, plates were incubated with 50 µl of Streptavidin-HRP (1:1000 in PBS-1% BSA) 1 h at RT. Wells were washed again 3 times and then 50 µl of OPD (o-phenylenediamine dihydrochloride) substrate (Sigma) added. The reaction was stopped by adding 50 µl of 2M H_2_SO_4_ and absorbance at OD_492_ was measured in a FLUO Star Omega Microplate Reader (BMG Labtech). Positive and negative controls were included in all the plates.

### Confocal Microscopy

FACS isolated HK IgM^+^/CD38^-^ and IgM^+^/CD38^+^ B cell populations were collected as described above and seeded on a poly-L-lysine (0.01% solution, Sigma)-coated slides and incubated at RT for 30 min in a humidified chamber. The slides were then fixed in 4% paraformaldehyde solution for 30 min at RT. The fixed samples were incubated for 1 h at RT with a blocking solution (TBS, pH 7.5 containing 5% BSA and 0.5% saponin) to permeabilize the cells and to minimize non-specific adsorption of the antibodies to the coverslip. The samples were then incubated with a mAb against trout IgM coupled to APC (17 mg/ml) for 1 h at RT in a humidified chamber. Slides were counterstained with 1 μg/ml DAPI (Sigma-Aldrich) for 10 min at RT, rinsed with PBS 1x and mounted with Fluoromount (Sigma-Aldrich) for microscopy. Laser scanning confocal microscopy images were acquired with an inverted Zeiss Axiovert LSM 880 microscope with Zeiss Zen software. Images were analyzed and processed with Zeiss Zen and Adobe Photoshop CS6 software packages.

### Statistical Analysis

Data handling, analysis and graphic representation were performed using GraphPad Prism version 7.00 for Windows, GraphPad Software, La Jolla California USA (www.graphpad.com). Statistical analyses were performed using a two-tailed Student’s *t* test and the differences between the mean values were considered significant when *P* ≤ 0.05.

## Results

### Evolution of ADP-Ribosyl Cyclase/Cyclic ADP-Ribose Hydrolase Proteins

**Figure 1:**
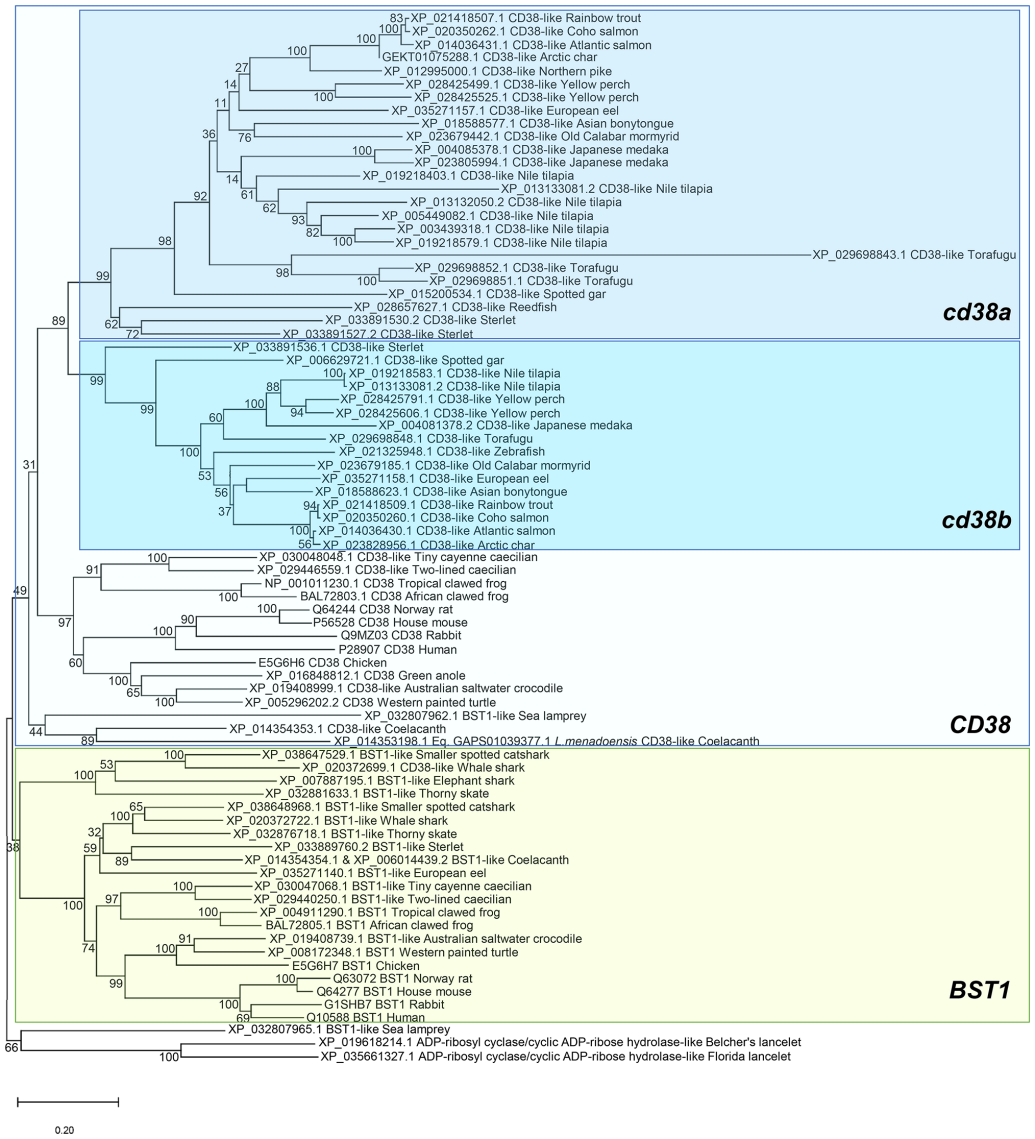
Phylogenetic tree using BST1 and CD38 proteins from the genomes of several species included in the RefSeq databases. Protein alignments were performed using the ClustalW software and a phylogenetic tree constructed using maximum likelihood analysis. Tree confidence was tested using bootstrapping analysis (1,000 replicates). ADP-ribosyl cyclase/cyclic ADP-ribose hydrolase proteins identified in lancelets (*Branchiostoma belcherii* and (*B. floridae*) were included as outgroup and used for rooting purposes.

To study the evolution of *CD38* (ADP-ribosyl cyclase/cyclic ADP-ribose hydrolase 1) and *BST1* (ADP-ribosyl cyclase/cyclic ADP-ribose hydrolase 2), homologue genes were identified along the genomes of several species covering key groups throughout species evolution. Multiple protein alignments were undertaken followed by a phylogenetic tree reconstruction. Starting the analysis in cephalochordates, both in the Belcher and in Florida lancelet genome, unique genes annotated as “ADP-ribosyl cyclase/cyclic ADP-ribose hydrolase” were identified, which were used as a root in the phylogenetic tree (**Figure 1**). A first evidence of gene duplication was detected in the sea lamprey genome, where two genes annotated as ADP-ribosyl cyclase/cyclic ADP-ribose hydrolase 2-like (*BST1*-like) were identified. The first of these genes was close in the phylogenetic tree to the lancelet proteins, whereas the second one appeared closely related with genes annotated as *CD38* (**Figure 1**). Following evolution, this duplication of *BST1*-like genes is conserved in several genomes from chondrichthyes, including smaller spotted catshark and thorny skate whereas other species like elephant shark only conserve one gene copy. In general, no genes annotated as *CD38*-like were identified in the genomes of these species. Interestingly, three genes encoding ADP-ribosyl cyclase/cyclic ADP-ribose hydrolase proteins were identified in coelacanths, key species in fish evolution. Among these three genes, one was grouped with *BST1*-like genes whereas the other two genes annotated as *CD38*-like were grouped together within the *CD38* group. Remarkable differences were observed when tetrapods and fish species were compared. In general, the genomes from tetrapod species (including frogs, reptiles, birds and mammals) present one gene encoding CD38 and another gene encoding BST1, which form different subgroups in the phylogenetic tree. Fish genomes in general do not contain BST1-like genes, with the exception of sterlet and European eel genomes, which present homologue genes located in the phylogenetic tree together with *BST1*-like genes from chondrichthyes. In contrast, several fish genomes contain at least two genes encoding *CD38*-like genes, designated as *cd38a* and *cd38b*, which form two independent clusters in the phylogenetic tree (**Figure 1**). This is the case for rainbow trout in which *cd38a* (XP_021418507.1) and c*d38b* (XP_021418509.1) genes were identified. In some species, a significant gene expansion has occurred, as three copies have been identified in sterlet and Japanese medaka, four in yellow perch and torafugu, and eight copies in Nile tilapia.

**Figure 2:**
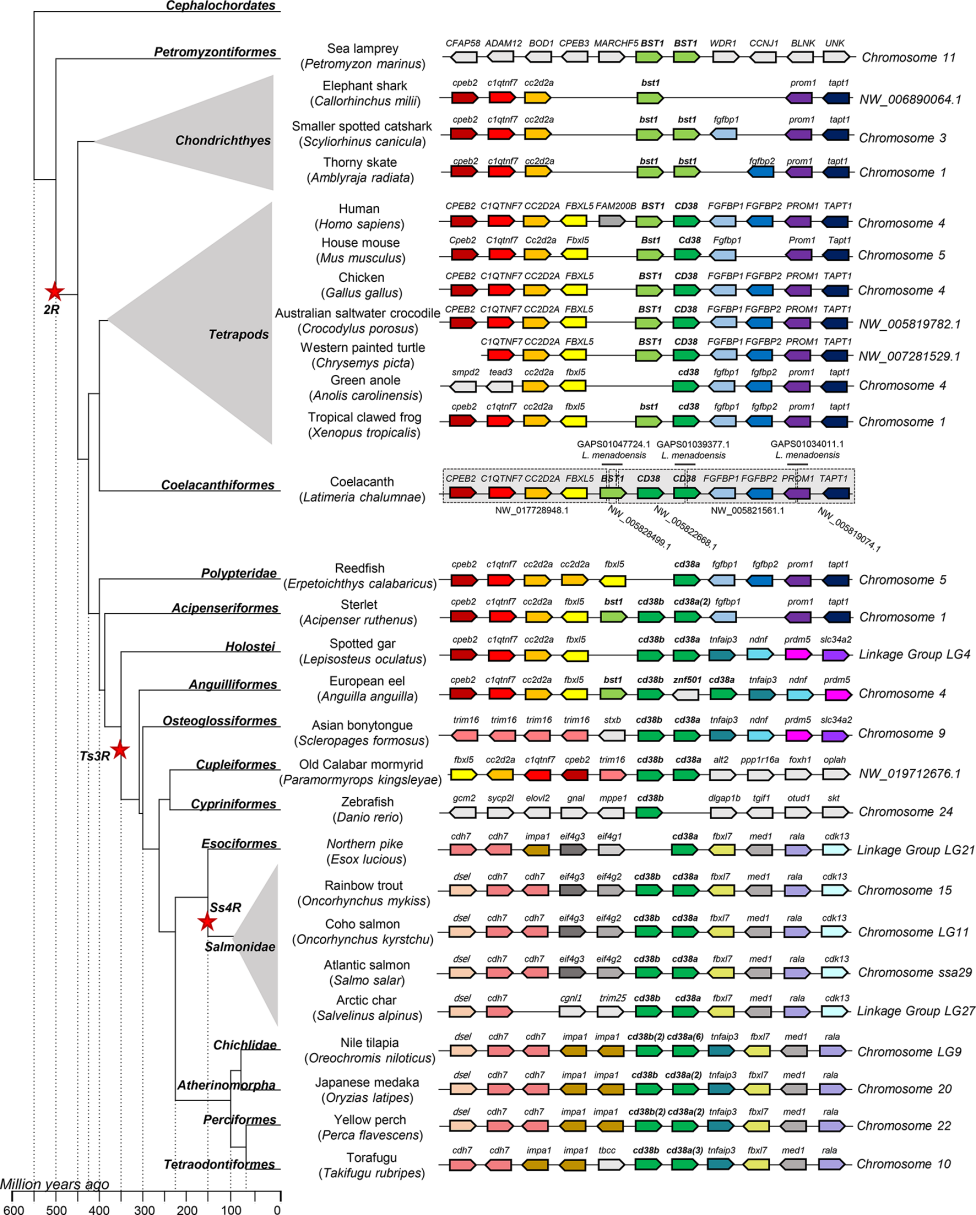
Synteny analysis of *BST1* and *CD38* genes associated to phylogenetic reconstruction. Different genes showing certain degree of conservation along species are represented by different coloured arrows. Non-conserved genes along the figure are showed in light grey. The neighbouring genes of *BST1-CD38* were identified along different genomes available in the RefSeq database. Arrows indicate the gene direction relative to *CD38.*Coelacanth genome showed partial sequences in the region of interest, thus this region was reconstructed from several scaffolds (grey boxes) using sequence information from ESTs identified in *Latimeria menadoensis* (TSA database from Genbank) which were used for guided assembly of different scaffolds (black lines).

A synteny analysis was performed to explore the genomic context of the identified *CD38* or *BST1* homologues genes. As mentioned above, the lancelet genome presents only one gene whereas the two copies of sea lamprey *BST1*-like genes are located in tandem in chromosome 11 (**Figure 2**). The genomic context of these genes in both species is exclusive and very different from that of other classes. Analyzing the chondrichthye genomes, two gene copies of *BST1*-like genes from the smaller spotted catshark and thorny skate are also located in tandem (**Figure 2**). In tetrapods, most species contain one *BST1* and one *CD38* gene, which are also found in tandem (**Figure 2**). Interestingly, all neighboring genes identified in chondrichthyes are conserved in tetrapods and also in some ancient fish, in which some new genes appear, such as *FBXL5* near *BST1* or either *FGFBP1* and *FGFBP2* near *CD38* (**Figure 2**). Total conservation of synteny was found between tetrapods and coelacanths as well as in the ancient fish species redfish and sterlet (**Figure 2**). In addition, a partial synteny conservation maintaining the same genes at one side of the studied genes was identified in spotted gar or European eel (**Figure 2**). Synteny conservation was also observed in a more evolved teleost fish species such as old calabar mormyrid, although in this case a global inversion was observed (**Figure 2**). Although synteny is not conserved between highly evolved teleost fish and tetrapods, the gene *tnfaip3* located near *cd38a* in some ancient fish is also conserved in the same position and orientation in several more evolved teleost fish such us Japanese medaka, yellow perch, torafugu, and Nile tilapia, species that then show a high degree of synteny conservation at both sides of the genes of interest with Northern pike and salmonids (**Figure 2**).

### Secondary and 3D Structure Analysis of ADP-Ribosyl Cyclase/Cyclic ADP-Ribose Hydrolase Proteins

The amino acid sequences of rainbow trout CD38A and CD38B were analyzed to identify structural features and model their potential 3D structure. According to the initial Blastp results both proteins showed CD38 as the most homologue human protein with aa identities of 34.01 and 34.68, respectively. The predicted secondary structure is highly similar for both proteins with a 42-47% of the sequence predicted to be alpha helixes and 12% predicted to form beta strands (**Figure S2**). Both proteins also showed a transmembrane helix between amino acids 142-151 in CD38A and 126-135 in the case of CD38B (**Figure S2**). These structural similarities were confirmed by the 3D structure homology modelling (**Figure S2**).

### Constitutive Expression of CD38A and CD38B in Rainbow Trout Tissues and IgM^+^ B Cells

**Figure 3:**
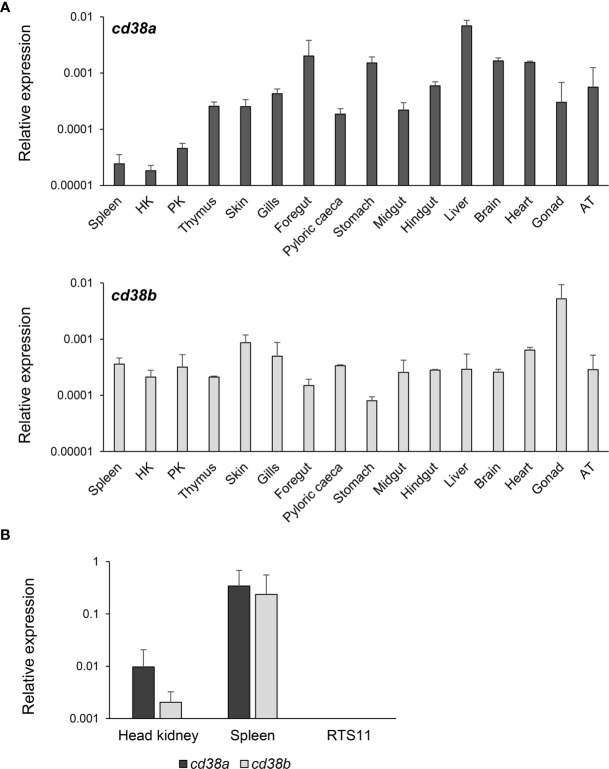
Constitutive levels of transcription of CD38A and CD38B. **(A)** The levels of transcription of CD38A and CD38B was estimated in spleen, HK, posterior kidney (PK), thymus, skin, gills, foregut, pyloric caeca, stomach, midgut, hindgut, liver, brain, heart, gonad and adipose tissue (AT) obtained from 3 non-stimulated perfused fish through real time PCR. Data are shown as the mean gene expression relative to the expression of endogenous control EF-1α + SD (n=3). **(B)** The levels of transcription of CD38A and CD38B were also estimated in isolated IgM^+^ B cells from spleen and HK, and compared to those observed in the RTS11 cell line. Data are shown as the mean gene expression relative to the expression of endogenous control EF-1α + SD (n=5).

CD38A was constitutively transcribed in all rainbow trout tissues analysed (**Figure 3A**). Transcription levels were highest in foregut, stomach, liver, brain and heart, and lowest in spleen, head and posterior kidney (**Figure 3A**). In the case of CD38B, transcription levels were highest in skin, gills, heart and gonad, whereas slightly lower mRNA levels were observed in stomach (**Figure 3A**).

The levels of transcription of CD38A and CD38B were also studied in isolated IgM^+^ B cells from kidney or spleen. Both populations constitutively transcribed CD38A and CD38B, but higher mRNA levels were detected for both genes in spleen IgM^+^ B cells than in kidney IgM^+^ B cells (**Figure 3B**). No CD38A or CD38B transcription was detected in the monocyte-macrophage cell line RTS11 (**Figure 3B**).

### Modulation of CD38A and CD38B Transcription in Response to a Viral Infection

**Figure 4:**
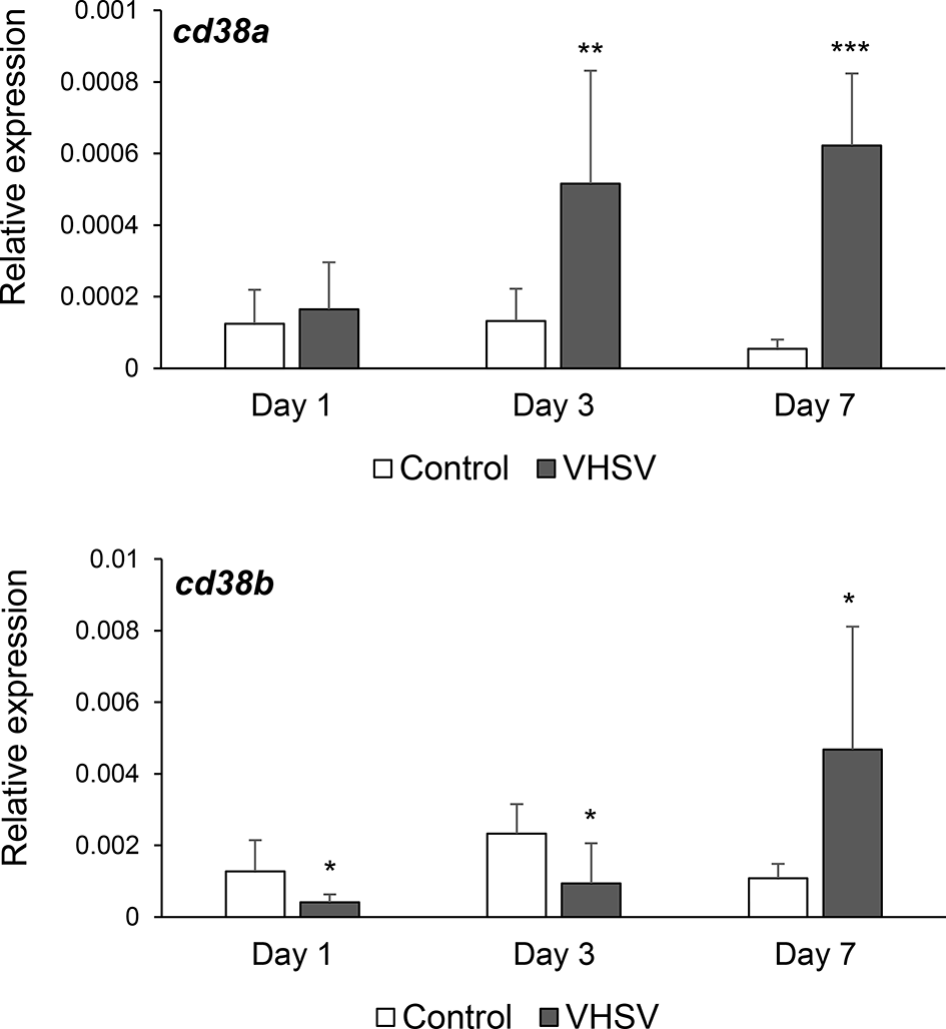
Effect of VHSV infection on CD38A and CD38B transcription levels in kidney. Rainbow trout were bath infected with VHSV (5 × 10^5^ TCID_50_/ml) or mock-infected. At days 1, 3 and 7 post-infection six trout from each group were euthanized and the kidney sampled to determine the levels of expression of CD38A and CD38B genes by real-time PCR. Data are shown as the mean gene expression relative to the expression of endogenous control EF-1α + SD. Asterisks denote levels of expression significantly different to those observed in mock-infected fish (*P ≤ 0.05, **P ≤ 0.01 and ***P ≤ 0.001).

To establish whether CD38A and CD38B transcription levels could be modulated in response to a pathogenic encounter, we infected fish with VHSV and determined the levels of transcription of both genes in the HK, an organ known to contain B cells in different stages of maturation/differentiation, including plasmablasts and PCs (25). For this, we challenged rainbow trout with VHSV by bath and sampled the HK after 1, 3 or 7 days of infection. CD38A transcription was significantly up-regulated at days 3 and 7 post-infection (**Figure 4**). In contrast, CD38B mRNA levels were significantly down-regulated in response to the virus at days 1 and 3 post-infection, but then increased significantly in comparison to the levels obtained in control fish at day 7 post-infection (**Figure 4**). It should be noted that these changes in CD38A and CD38B transcription could imply regulation of mRNA levels in cells, or mobilization of cells expressing CD38A or CD38B from or to the HK.

### Production of a Specific Anti-CD38A Antibody

**Figure 5:**
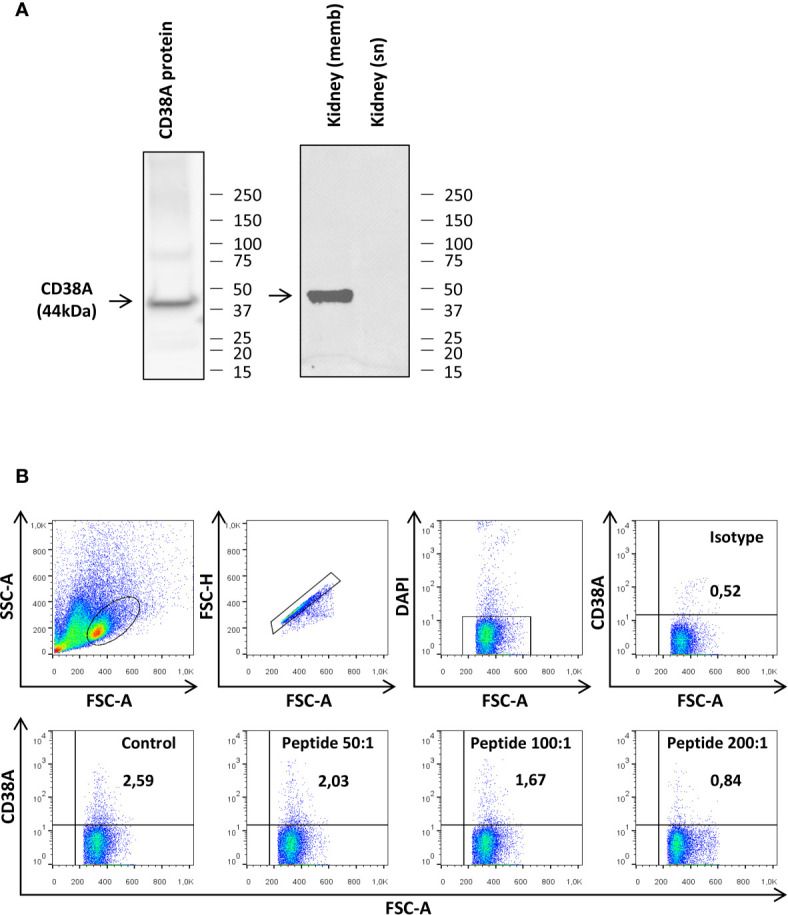
Testing of the specificity of the anti-CD38A mAb. **(A)** The specificity of the anti-CD38A mAb was demonstrated by Western blot analysis using the recombinant CD38 protein used for immunization and kidney protein lysates. Detection of CD38A was compared in lysates enriched in cell membranes (memb) and lysates enriched in soluble proteins (sn) **(B)** The specificity of the mAb was also established by flow cytometry, blocking the antigen recognition site of the anti-CD38 mAb by pre-incubation with the recombinant CD38 protein. The results obtained in a representative spleen culture are shown, including the plots obtained when cells were incubated with the isotype control, with the preincubated mAb at different peptide:mAb ratios (200:1. 100:1 and 50:1) or with the mAb alone (Control).

To establish whether CD38A was a marker for specific B cell subsets as in mammals, we raised a mAb to a recombinantly produced extracellular domain of the rainbow trout CD38A. The specificity of the different clones was initially tested by ELISA (data not shown) and Western blot using this recombinant protein. Thus, clone 6E4G8 seemed to specifically recognize a protein of the expected size (44 KDa corresponding to 31 KDa of extracellular CD38A and 13 KDa of the histidine tag) by Western blot (**Figure 5A**). The specificity of the Ab was then tested by Western blot using rainbow trout kidney lysates. 6E4G8 recognized a band of the expected size (45 KDa corresponding to the complete CD38A protein) in kidney lysates enriched in cell membranes but did not detect the protein in kidney lysates enriched in soluble proteins (**Figure 5A**).

We next tested whether the mAb raised against CD38A was capable of detecting CD38A on the cell surface by flow cytometry. This initial experiment was performed using spleen leukocytes. Approximately 2.5% of splenocytes expressed CD38A on the cell membrane (**Figure 5B**). The binding specificity was also assessed by flow cytometry blocking the antigen recognition site with the recombinant protein used for the immunization. After the blockage of the mAb, only a residual population with non-specific binding could be detected (**Figure 5B**), indicating a specific recognition. Furthermore, this blockage was not observed when an irrelevant peptide was used (data not shown).

### Identification of IgM^+^ B Cell Subsets Expressing CD38A on the Membrane

**Figure 6:**
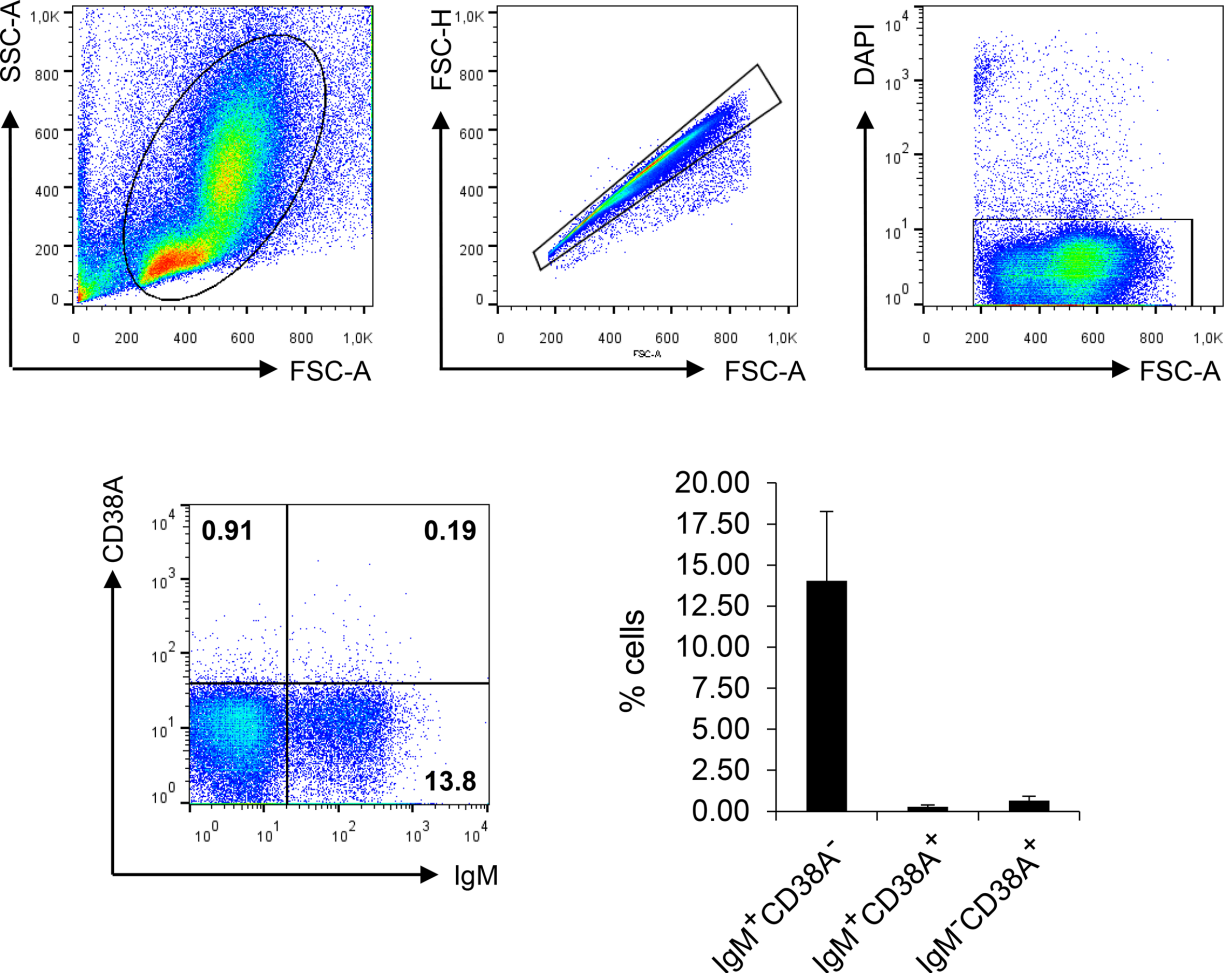
Analysis of HK leukocyte populations expressing CD38A on the cell surface. HK leukocytes were stained with DAPI and the mAbs against IgM and CD38A. For each sample, FSC/SSC profiles were used to define lymphoid gates. Single cells were identified by plotting FSC-H/FSC-A profiles. DAPI negative cells within the singlet gate were gated in order to select live cells. The percentage of IgM^+^CD38A^+^, IgM^+^CD38A^-^ and IgM^-^CD38A^+^ cells was then established among live singlet lymphoid cells. Representative dot plots are shown along with a graph showing the mean percentage of the different cell subsets + SD (n=6).

Having established the specificity of the rainbow trout anti-CD38A mAb, we used it to investigate CD38A surface expression on IgM^+^ B cells from the HK. We detected a small subpopulation of IgM^+^CD38A^+^ B cells that corresponded to approximately 0.2% of all kidney leukocytes, accounting for approximately 1.9% of all IgM^+^ B cells found in this tissue (**Figure 6**). Cells expressing CD38A on the cell surface but no IgM were also detected in the cultures, accounting for approximately 0.4% of all kidney leukocytes (**Figure 6**). Similarly, IgM^+^CD38A^+^ B cells and IgM^-^CD38A^+^ cells were also detected in blood, spleen and even in mucosal tissues such as gills (**Figure S3**). Interestingly, in the gills, the percentage of IgM^-^CD38A^+^ cells was much higher than in systemic tissues, accounting for approximately 3.25% of the total leukocyte population. The nature of these cells is still unknown.

### Regulation of IgM^+^CD38A^+^ B Cells in Response to Different Stimuli

**Figure 7:**
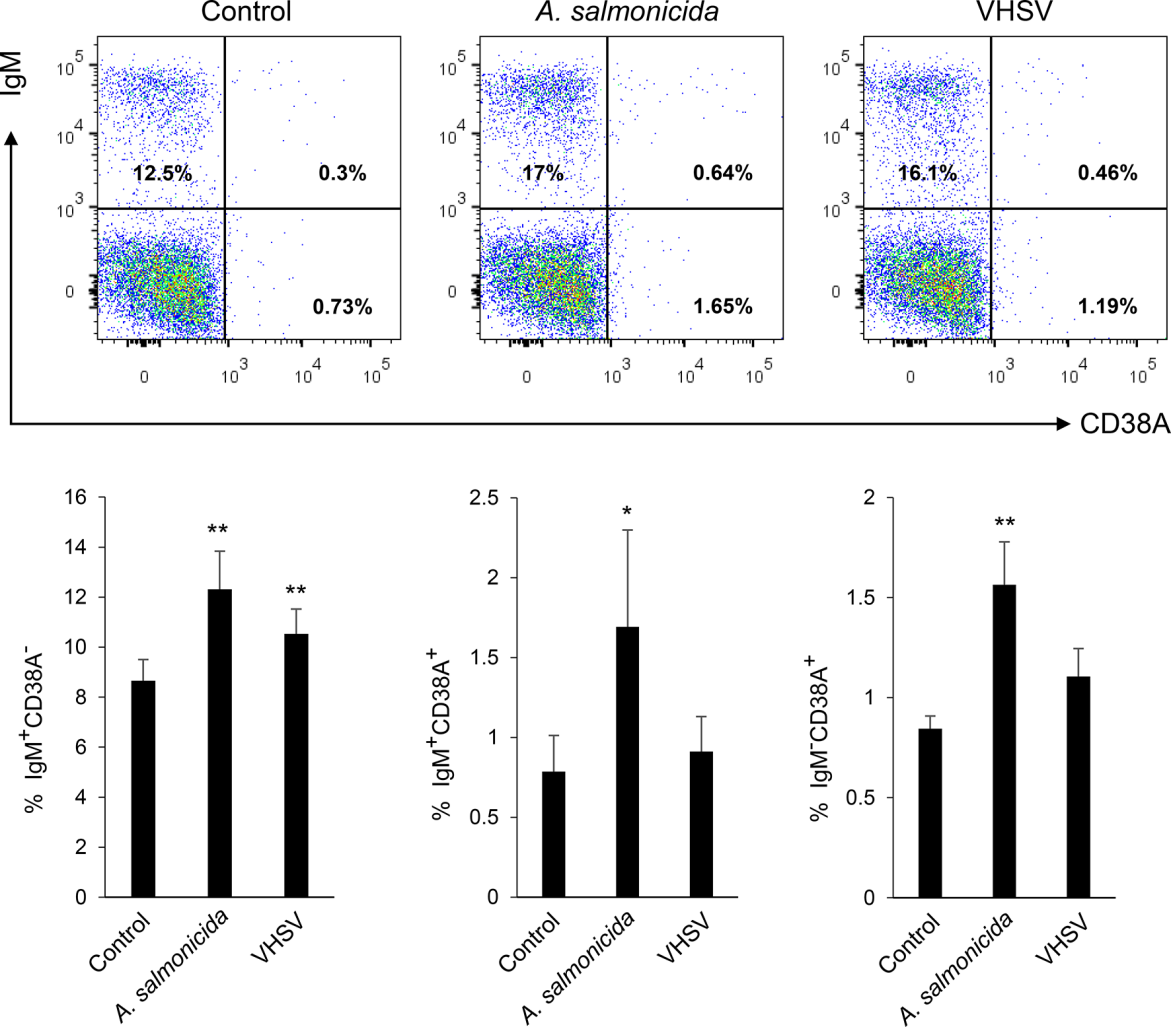
Determination of IgM^+^CD38A^+^, IgM^+^CD38A^-^ and IgM^-^CD38A^+^ cell populations in HK leukocyte cultures after exposure to different stimuli. HK leukocytes were exposed to inactivated *Aeromonas salmonicida* (2 x 10^6^ bacteria/ml) or inactivated VHSV (1 x 10^6^ TCID_50_/ml). Cells incubated with media alone were also included. After 72 h of incubation at 20°C, cells were stained with anti-IgM and anti-CD38A and analysed by flow cytometry as described in the legend of **Figure 6**. Representative dot plots are shown along with graphs showing the mean percentage of the different cell subsets + SD (n=10). Asterisks denote percentages significantly higher than those observed in untreated cultures (*P ≤ 0.05 and **P ≤ 0.01).

To determine if the number of cells expressing CD38A could be regulated in HK B cells in response to an immune stimulus, we exposed HK leukocyte cultures to a viral or a bacterial antigen and then studied the expression of CD38A on IgM^+^ B cells through flow cytometry. After 72 h of stimulation, only *A. salmonicida* was capable of significantly up-regulating the percentage of IgM^+^CD38A^+^ cells in the cultures, whereas VHSV on the contrary, had no effect (**Figure 7**). As previously reported (40, 41), the percentage of IgM^+^ B cells with no CD38A on the membrane (IgM^+^CD38A^-^ cells) increased significantly in response to both stimuli (**Figure 7**). Finally, the percentage of CD38A^+^ cells with no IgM on the membrane (IgM^-^CD38A^+^ cells) also increased in response to *A. salmonicida* (**Figure 7**).

### Characterization of HK IgM^+^CD38A^+^ B Cells

**Figure 8:**
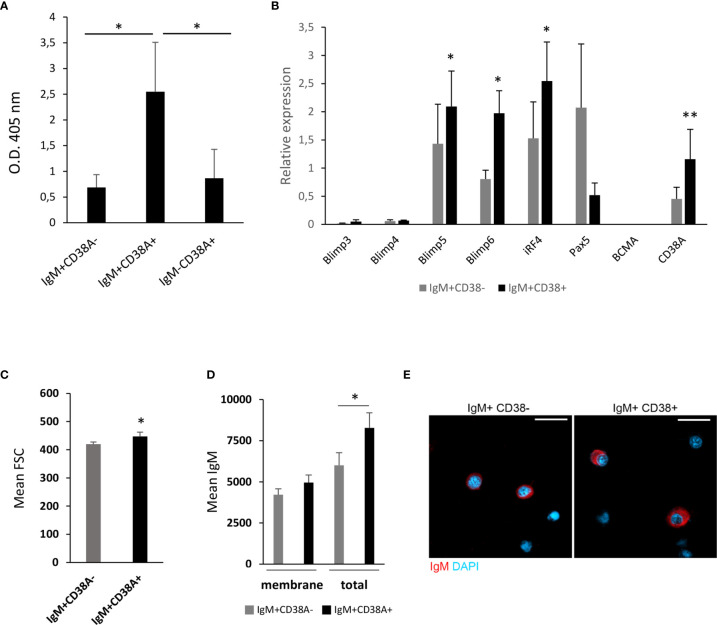
Kidney IgM^+^CD38A^+^ cells correspond to cells with an increased capacity to secrete IgM. **(A)** Kidney IgM^+^CD38A^+^, IgM^+^CD38A^-^ and IgM^-^CD38A^+^ cells isolated by flow cytometry using a BD FACSAria III cell sorter were seeded in 96-well plates and incubated at 20°C for 3 days. After this time, supernatants were collected to determine total IgM concentration in supernatants by ELISA. Results are shown as mean absorbance at 405 nm + SD (n=5) **(B)** The levels of transcription of genes involved in the differentiation of B cells to plasmablasts/PCs were also evaluated through real time PCR in IgM^+^CD38A^+^ and IgM^+^CD38A^-^ B cell populations. Data are shown as the mean gene expression relative to the expression of endogenous control EF-1α + SD (n=8). **(C)** Quantification of FSC MFI values of IgM^+^CD38A^+^ and IgM^+^CD38A^-^ B cell populations, shown as mean + SEM (n=7). **(D)** Quantification of membrane IgM and total (membrane plus intracellular) IgM MFI values of IgM^+^CD38A^+^ and IgM^+^CD38A^-^ B cell populations, shown as mean + SEM (n=6). **(E)** Visualization of FACS sorted head kidney IgM^+^CD38^-^ and IgM^+^CD38^+^ B cells. Examples of each subset are shown. Scale bars: 5 μm. Asterisks denote significantly different values in IgM^+^CD38A^+^ and IgM^+^CD38A^-^ B cell populations or between indicated groups (*P ≤ 0.05 and **P ≤ 0.01).

In order to provide some insights on the nature of these IgM^+^ B cells that expressed CD38A on the membrane, we sorted IgM^+^CD38A^+^, IgM^+^CD38A^-^ B cells and IgM^-^CD38A^+^ cells and then analysed their IgM secreting capacity. We established that the amount of IgM secreted by isolated IgM^+^CD38A^+^ B cells after 3 days in culture was significantly higher than that produced by IgM^+^CD38A^-^ B cells (**Figure 8A**). The capacity of IgM^-^CD38A^+^ cells to secrete IgM was quite similar to that of IgM^+^CD38A^-^ B cells and significantly lower than that of IgM^+^CD38A^+^ B cells (**Figure 8A**).

Having established the superior capacity of IgM^+^CD38A^+^ B cells to secrete IgM, we studied in these cells the levels of transcription of a selection of genes implicated in the differentiation of B cells to plasmablasts/PCs, comparing them to those obtained in IgM^+^CD38A^-^ B cells. Our results revealed that IgM^+^CD38A^+^ B cells have significantly higher mRNA levels of two homologues of mammalian Blimp1 (*prdm1-c1* and *prdm1-c2*) and *irf4* in comparison to IgM^+^CD38A^-^ B cells (**Figure 8B**). Additionally, the levels of *pax5* transcription were lower in IgM^+^CD38A^+^ B cells when compared to IgM^+^CD38A^-^ B cells, although in this case the differences were not significant due to a high individual variability (**Figure 8B**). Nevertheless, the transcriptional profile of IgM^+^CD38A^+^ B cells agrees with that of cells that have started a differentiation process to plasmablasts/PCs. To further support this statement, we verified that IgM^+^CD38A^+^ B were significantly larger than of IgM^+^CD38A^-^ B cells (**Figure 8C**). IgM^+^CD38A^+^ B cells also showed more total IgM (both membrane and intracellular IgM) than IgM^+^CD38A^-^ B cells (**Figure 8D**). Finally, visualization of the sorted B cell subsets also evidenced that IgM^+^CD38A^+^ B cells had a larger cytoplasm-to-nucleus ratio than did IgM^+^CD38A^-^ B cells (**Figure 8E**). All of these features support the hypothesis that B cells expressing CD38A on the membrane have undergone a differentiation process to plasmablasts/PCs.

## Discussion

The important differences that exist between fish and mammalian immune structures and existing elements, make it difficult to assume that fish B cells will be regulated as mammalian conventional B cells. In fact, recent evidence points to a functional and phenotypical resemblance of fish B cells with mammalian B1 cells (12, 42, 43). Nevertheless, whether different B cell subsets coexist in teleost fish as in mammals is still unknown, apart from those B cell subsets defined by the pattern of expression of Igs on the cell membrane (IgM^+^IgD^+^, IgM^+^IgD^-^, IgD^+^IgM^-^ and IgT^+^ B cells). Similarly, tools to differentiate among B cells in different stages of differentiation are also lacking, strongly hampering a deeper understanding of how teleost B cells are regulated.

Although CD38 is broadly expressed in many different immune and non-immune cell types, it has been used extensively to classify various subpopulations of lymphocytes in both humans and mice (23, 24). However, the pattern of expression of CD38 throughout the B cell lineage significantly differs in mice and human. In mice, highest CD38 are found in immature B cells emerging from bone marrow, splenic T2 lymphocytes, MZ B and B1 cells [reviewed in (23)]. In contrast, in humans, terminally differentiated plasma cells express the highest levels of surface CD38 (24). In this context, we searched for CD38 homologues in rainbow trout and studied whether they could be also used as markers to differentiate between B cell subsets in this species.

In mammals, two closely related ADP-ribosyl cyclase/cyclic ADP-ribose hydrolase proteins have been described, namely CD38 and BST1. In these genomes, the genes encoding for these proteins are found in tandem. Although chondrichthyes only contain *BST1*-like genes, three genes encoding ADP-ribosyl cyclase/cyclic ADP-ribose hydrolase proteins, were identified in coelacanths, key species in fish evolution. Among these three genes, one was grouped with *BST1*-like genes whereas the other two genes were grouped together within the *CD38* group. Teleost fish, however, do not contain *BST1*-like genes, but contain one or several copies of *CD38* genes located consecutively in the same chromosome. This tandem duplication activity was found to be especially intense in some fish species which have acquired a large repertoire of *CD38*-like genes. The fact that other genes located in this region have also suffered several duplications seems to reflect a high propensity of this locus to experience this type of duplications.

In rainbow trout, two *CD38*-like genes were identified, which we designated as *cd38a* and *cd38b*. Thus, we first performed a transcriptional study to establish the pattern of expression of these genes in homeostasis and in response to an immune stimulus. Both genes were constitutively expressed in all tissues analyzed and up-regulated in response to a viral infection, but some differences were observed suggesting that the regulation and possibly the role of these two homologues might have diverged.

Both CD38A and CD38B were found to be constitutively transcribed by rainbow trout spleen and HK IgM^+^ B cells. In Nile tilapia, CD38 was shown to be expressed on the cell membrane of HK IgM^+^ B cells through immunofluorescence by use of a polyclonal antibody (44). However, in that study, the percentage of IgM^+^ B cells that expressed CD38 on the cell membrane was not specified. In rainbow trout, we identified two IgM^+^ B cell populations according to whether they expressed or not CD38A on the cell membrane, namely IgM^+^CD38A^+^ and IgM^+^CD38A^-^ B cells. Although this percentage of IgM^+^ B cells that expressed CD38A on the cell membrane was low in homeostasis, we have also shown that stimulation with *A. salmonicida* can significantly increase this number. Previous studies from our group had shown that rainbow trout leukocyte cultures significantly increased the number of ASCs when *in vitro* stimulated with *A. salmonicida* (41), so the increase in the number of CD38A^+^ subpopulations in response to the bacteria was not unexpected. Similarly, tilapia CD38 mRNA levels were up-regulated in response to LPS in HK leukocytes stimulated *in vitro* (44).

Although the exposure of HK leukocytes to inactivated VHSV *in vitro* was not capable of significantly increasing the percentage of IgM^+^ B cells expressing CD38A on the cell membrane nor that of cells expressing CD38A without IgM, a bath infection with VHSV significantly up-regulated the levels of transcription of both CD38 homologues in the HK. The fact that additional signals generated by exposure to a live virus are required for IgM^+^ B cells to increase CD38A expression levels in response to a virus, could explain why these up-regulations were observed *in vivo* but not *in vitro* in response to the inactivated virus. This is an issue that should be further explored in future experiments. Nevertheless, our results have revealed an important role of CD38 in the response to VHSV infection. In mammals, CD38 has also been shown to have an important role in the control of different infectious diseases. Thus, CD38 deficiency increased the susceptibility of mice to bacteria such as *Listeria monocytogenes* (45), *Mycobacterium avium* (46) and *Streptococcus pneumoniae* (47) or parasites like *Entamoeba histolytica* (48). Furthermore, a number of studies have shown the importance of CD38 in HIV infection (49). Hence, in HIV patients, CD38 is up-regulated in CD4^+^ and CD8^+^ T cells early after HIV infection (50) and the percentage of CD8^+^CD38^+^ T cells is decreased in patients that have been treated with antiretrovirals (51). Regarding B cells, CD38 also has been shown to play an important role in B cell functionality in mammals, surely conditioning the response to pathogens. Thus, CD38 ligation by a monoclonal antibody was shown to prevent apoptosis of GC B cells, thereby affecting the selection of B cells with high affinity at these sites (52). Splenic B cells from mice were also shown to differentiate to IgM secreting cells upon stimulation with anti-CD38 and IL-5 (53).

In the current study, we have demonstrated that IgM^+^ B cells expressing CD38A on the cell membrane (IgM^+^CD38A^+^ B cells) secreted significantly higher levels of IgM in culture than IgM^+^CD38A^-^ B cells. Additionally, when we evaluated the transcription of several differentiation markers in both populations, we found that IgM^+^CD38A^+^ B cells expressed significant higher levels of two Blimp1 homologues (*prdm-c1* and *prdm1-c2*) and *irf4* compared to IgM^+^/CD38^-^ B cells. These cells were also larger, had more total IgM (both membrane and intercellular IgM) and had a larger cytoplasm-to-nucleus ratio than B cells with no CD38A on the membrane. These results strongly suggest that IgM^+^CD38A^+^ cells are at least partially differentiated towards a plasmablast/PC state. Similarly, three different B cell subsets were isolated in Nile tilapia according to their density. Among these subsets, the one that seemed to correspond to plasmabasts/PCs had significant higher transcription levels of CD38 and Blimp1 and lower mRNA levels of Pax5 and membrane IgM in comparison to resting mature B cells or partially activated B cells (44). Interestingly, IgM^-^ cells that expressed CD38A on the cell membrane were also identified in rainbow trout lymphoid tissues. However, the IgM secreting capacity of these cells was similar to that of resting IgM^+^ B cells. Thus, whether these cells correspond to IgT^+^ B cells or another leukocyte type should be further investigated in the future.

In conclusion, in this work, we have characterized two rainbow trout CD38 homologues (CD38A and CD38B), and analyzed their transcription patterns in response to a viral infection. The increase in the transcription of both homologues in the HK in response to VHSV seems to indicate an important role of CD38 in the teleost antiviral response. Additionally, we have generated a monoclonal antibody against CD38A and used it to discern between IgM^+^ cells that expressed CD38A and those that do not. As occurs in humans and Nile tilapia (44), CD38A expression seems to identify B cells that have started a differentiation towards plasmablasts/PCs as rainbow trout IgM^+^CD38A^+^ cells had a higher capacity to secrete IgM, were larger and had a transcriptional profile consistent with a more differentiated state. Furthermore, the percentage of these differentiated CD38A^+^IgM^+^ B cells significantly increased in HK leukocyte cultures in response to *A. salmonicida*. Altogether, these results point to CD38A as a relevant marker for B cell activation in rainbow trout as in humans.

## Data Availability Statement

The datasets presented in this study can be found in online repositories. The names of the repository/repositories and accession number(s) can be found in the article/**Supplementary Material**.

## Ethics Statement

The animal study was reviewed and approved by INIA Ethics Committee.

## Author Contributions

PP identified the CD38 homologues and performed all the *in silico* analysis with help from TW. DM performed and analyzed most experiments with help from EM, IS, JH-J, BA, and PD-R. EM provided support with all flow cytometry experiments and performed the cell sortings. LR established the specificity of the anti-CD38A mAb. CT conceived the work and designed the experiments with help from IS, PD-R, and PP. CT and BA wrote the main body of the paper with contributions from all other authors. All authors contributed to the article and approved the submitted version.

## Funding

This work was supported by the European Research Council (ERC Consolidator Grant 2016 725061 TEMUBLYM) and by the *Comunidad de Madrid* (grant 2016-T1/BIO-1672).

## Conflict of Interest

The authors declare that the research was conducted in the absence of any commercial or financial relationships that could be construed as a potential conflict of interest.

## Publisher’s Note

All claims expressed in this article are solely those of the authors and do not necessarily represent those of their affiliated organizations, or those of the publisher, the editors and the reviewers. Any product that may be evaluated in this article, or claim that may be made by its manufacturer, is not guaranteed or endorsed by the publisher.
