# Supplementary material for: CD38 Defines a Subset of B Cells in Rainbow Trout Kidney With High IgM Secreting Capacities
Source: Front Immunol. 2021 Nov 30;12:773888. doi: 10.3389/fimmu.2021.773888 (PMC8669677; doi:10.3389/fimmu.2021.773888)
Supplement: Supplementary file 1 [file Presentation_1.pptx]

## Slide 1
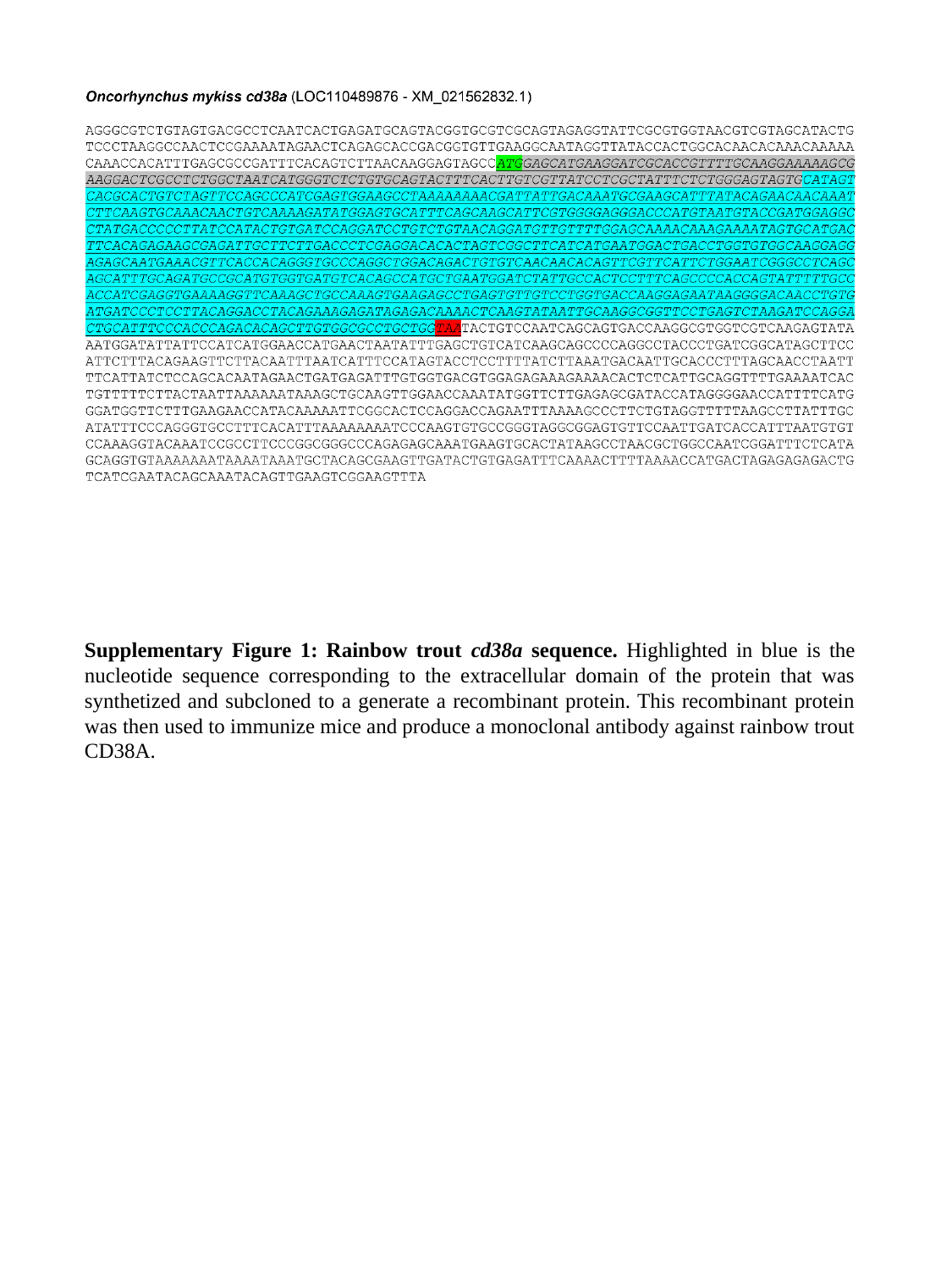

Supplementary Figure 1: Rainbow trout cd38a sequence. Highlighted in blue is the nucleotide sequence corresponding to the extracellular domain of the protein that was synthetized and subcloned to a generate a recombinant protein. This recombinant protein was then used to immunize mice and produce a monoclonal antibody against rainbow trout CD38A.

## Slide 2
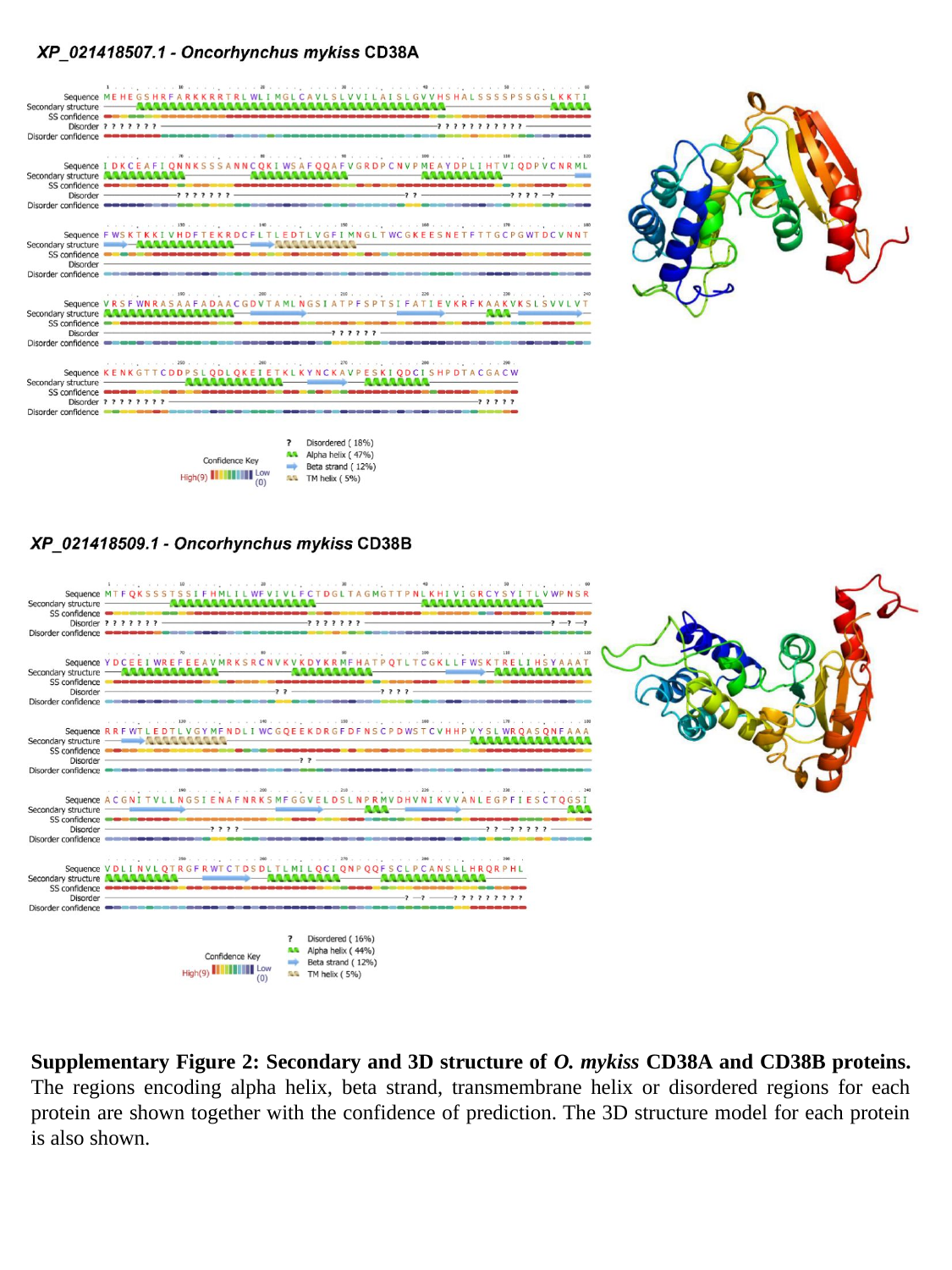

Supplementary Figure 2: Secondary and 3D structure of O. mykiss cd38a and cd38b proteins. The regions encoding alpha helix, beta strand, transmembrane helix or disordered regions for each protein are shown together with the confidence of prediction. The 3D structure model for each protein is also shown.

## Slide 3
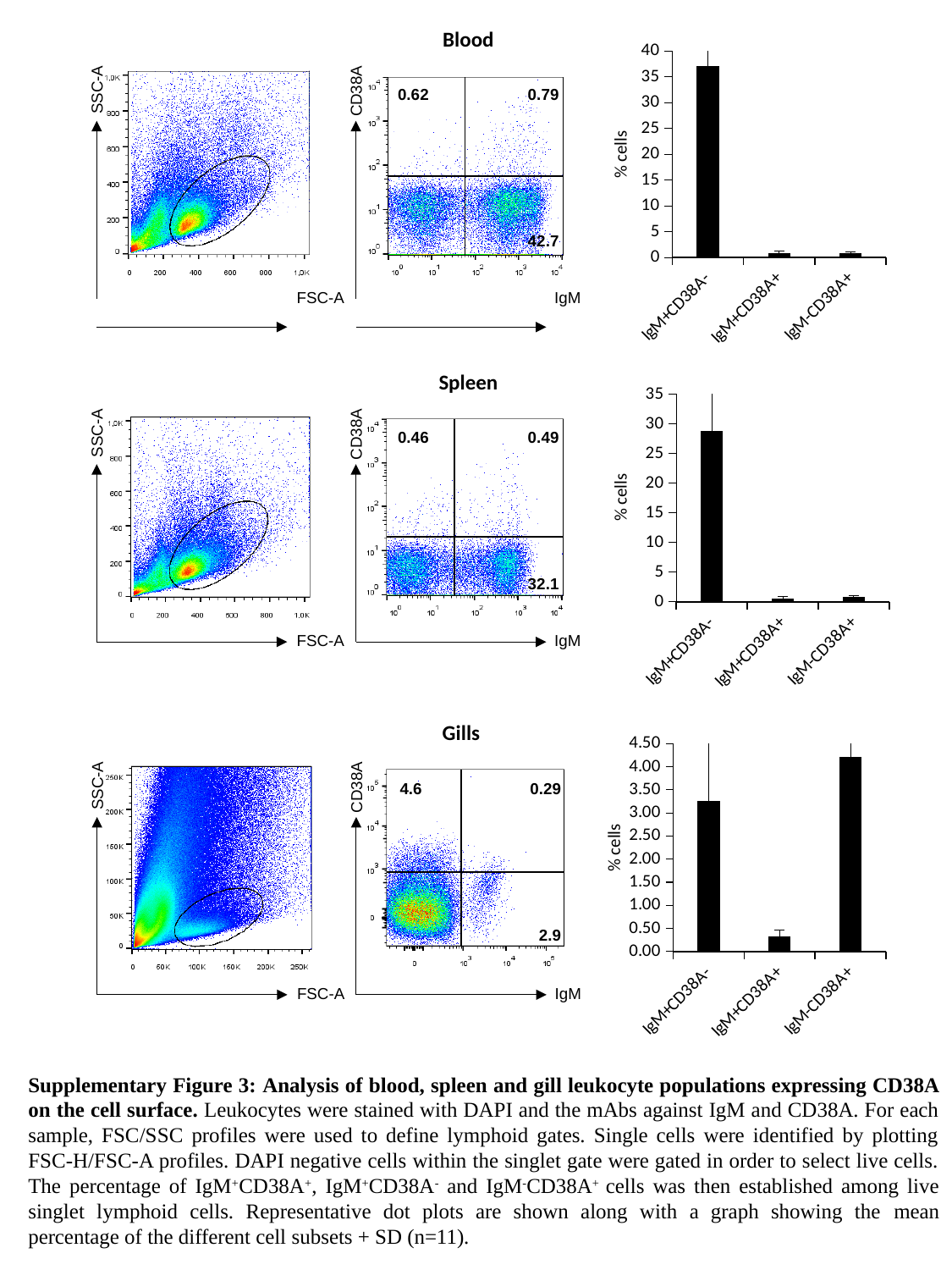

Blood
### Chart
| Category | |
|---|---|
| IgM+CD38A- | 37.1 |
| IgM+CD38A+ | 0.8527272727272727 |
| IgM-CD38A+ | 0.7872727272727271 |
0.62
0.79
SSC-A
CD38A
% cells
42.7
FSC-A
IgM
Spleen
### Chart
| Category | |
|---|---|
| IgM+CD38A- | 28.775454545454544 |
| IgM+CD38A+ | 0.5818181818181818 |
| IgM-CD38A+ | 0.7872727272727272 |
0.46
0.49
SSC-A
CD38A
% cells
32.1
FSC-A
IgM
Gills
### Chart
| Category | |
|---|---|
| IgM+CD38A- | 3.2536363636363634 |
| IgM+CD38A+ | 0.31736363636363635 |
| IgM-CD38A+ | 4.223636363636363 |
4.6
0.29
SSC-A
CD38A
% cells
2.9
FSC-A
IgM
Supplementary Figure 3: Analysis of blood, spleen and gill leukocyte populations expressing CD38A on the cell surface. Leukocytes were stained with DAPI and the mAbs against IgM and CD38A. For each sample, FSC/SSC profiles were used to define lymphoid gates. Single cells were identified by plotting FSC-H/FSC-A profiles. DAPI negative cells within the singlet gate were gated in order to select live cells. The percentage of IgM+CD38A+, IgM+CD38A- and IgM-CD38A+ cells was then established among live singlet lymphoid cells. Representative dot plots are shown along with a graph showing the mean percentage of the different cell subsets + SD (n=11).

## Slide 4
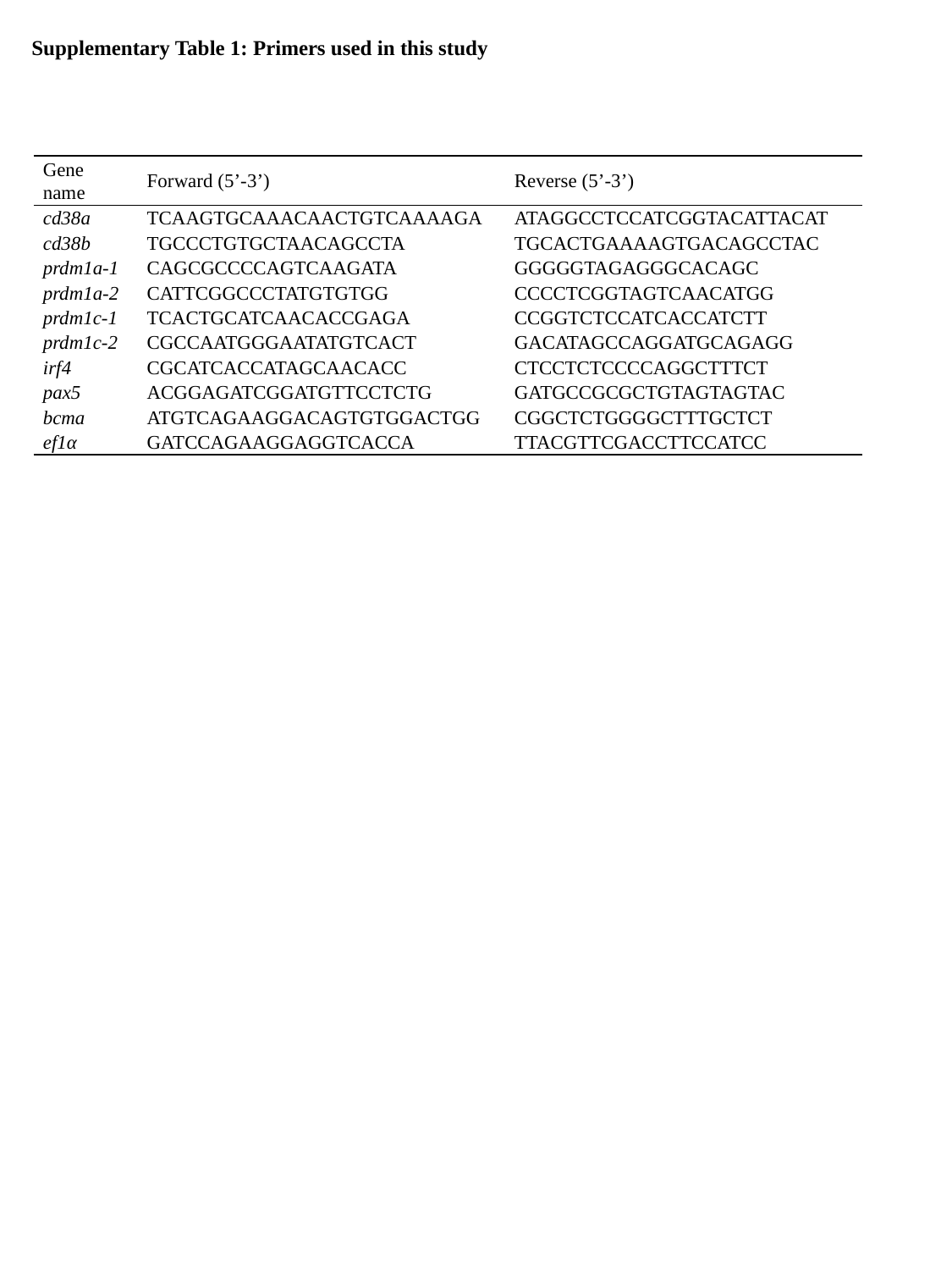

Supplementary Table 1: Primers used in this study
| Gene name | Forward (5’-3’) | Reverse (5’-3’) |
| --- | --- | --- |
| cd38a | TCAAGTGCAAACAACTGTCAAAAGA | ATAGGCCTCCATCGGTACATTACAT |
| cd38b | TGCCCTGTGCTAACAGCCTA | TGCACTGAAAAGTGACAGCCTAC |
| prdm1a-1 | CAGCGCCCCAGTCAAGATA | GGGGGTAGAGGGCACAGC |
| prdm1a-2 | CATTCGGCCCTATGTGTGG | CCCCTCGGTAGTCAACATGG |
| prdm1c-1 | TCACTGCATCAACACCGAGA | CCGGTCTCCATCACCATCTT |
| prdm1c-2 | CGCCAATGGGAATATGTCACT | GACATAGCCAGGATGCAGAGG |
| irf4 | CGCATCACCATAGCAACACC | CTCCTCTCCCCAGGCTTTCT |
| pax5 | ACGGAGATCGGATGTTCCTCTG | GATGCCGCGCTGTAGTAGTAC |
| bcma | atgtcagaaggacagtgtggactgg | CGGCTCTGGGGCTTTGCTCT |
| ef1α | GATCCAGAAGGAGGTCACCA | TTACGTTCGACCTTCCATCC |

## Slide 5
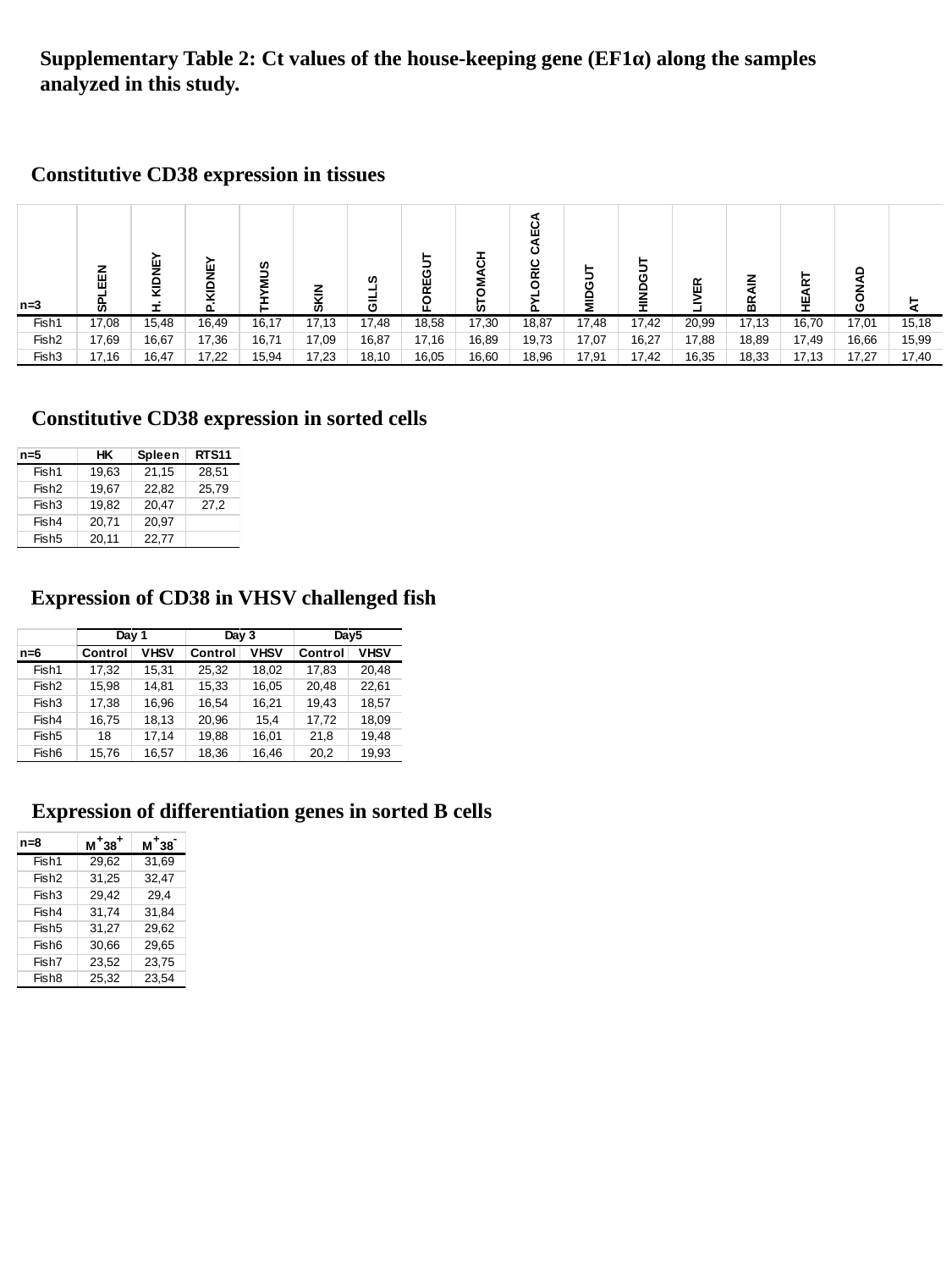

Supplementary Table 2: Ct values of the house-keeping gene (EF1α) along the samples analyzed in this study.
Constitutive CD38 expression in tissues
Constitutive CD38 expression in sorted cells
Expression of CD38 in VHSV challenged fish
Expression of differentiation genes in sorted B cells
